# Clinical implications of HLA locus mismatching in unrelated donor hematopoietic cell transplantation: a meta-analysis

**DOI:** 10.18632/oncotarget.15291

**Published:** 2017-02-11

**Authors:** Ruxiu Tie, Tiansong Zhang, Bo Yang, Huarui Fu, Biqing Han, Jian Yu, Yamin Tan, He Huang

**Affiliations:** ^1^ Bone Marrow Transplantation Center, The First Affiliated Hospital, College of Medicine, Zhejiang University, Hangzhou, China; ^2^ Department of Traditional Chinese Medicine, Jing'an District Central Hospital, Shanghai, China; ^3^ School of Public Health, Wenzhou Medical University, Wenzhou, China

**Keywords:** HLA locus, hematopoietic cell transplantation, unrelated donor, meta-analysis

## Abstract

It remains controversial that the impacts of individual HLA locus mismatches on clinical outcomes of patients receiving unrelated-donor hematopoietic cell transplantation (HCT), as compared to HLA allele matched controls. We conducted a meta-analysis to address these issues. Four databases (PubMed, Embase, Web of Science and the Cochrane Library) were searched to select eligible studies. All donor-recipient pairs were high-resolution typing for HLA-A, -B, -C, -DRB1, DQB1 and DPB1 loci. Multivariate-adjusted hazard ratios (HRs) were extracted and pooled using a random-effects model. A total of 36 studies were included, with 100,072 patients receiving HCT. Surprisingly, we found that HLA-DQB1 locus mismatches had no significantly increased risk of multiple outcomes including acute and chronic graft-versus-host disease (GVHD), overall mortality and disease relapse (HR, 1.07; *P* = .153; HR, 1.07; *P* = .271; HR, 1.09; *P* = .230; HR, 1.07; *P* = .142 and HR, 1.02; *P* = .806, respectively). Mismatched HLA-DPB1 was significantly associated with a reduced risk of disease relapse (HR, 0.74; *P* < .001) but not with increased risks of transplant-related mortality (TRM) and overall mortality (HR, 1.09; *P* = .591; I^2^ = 74.2% and HR, 1.03; *P* = .460, respectively). In conclusion, HLA-DQB1 locus mismatches is a permissive mismatching. HLA-DPB1 locus mismatches significantly protect against leukemia relapse. Refining effects of individual HLA locus mismatches contributes to predicting prognosis of patients receiving unrelated donor HCT.

## INTRODUCTION

During the past 36 years, unrelated donor HCT has already become one of the most effective but complex therapy for selected patients with hematologic malignancies or certain life-threatening nonmalignant disorders [1, 2]. However, the clinical application of the HCT is limited by leukemia relapse [3] and life-threatening complications, such as graft-versus-host disease (GVHD) [4, 5], infection [6–8], conditioning regimen-related toxicities [9–11], and transplant-associated thrombotic microangiopathy (TMA) [12–14] as well, which are more common in patients receiving HLA locus mismatched grafts. In clinical practice, it is increasingly difficult to identify a HLA locus completely matched donor in the presence of highly polymorphic HLA alleles. The most loci are the HLA class I (A, B and C) and the class II (DRB1, DQB1 and DPB1) molecules [15].

A large number of studies assessed the impact of individual HLA mismatches on multiple clinical outcomes [16–51]. For a given end point, the risk of a specific HLA locus mismatches was generally inconsistent or even contradictory across studies. These discrepancies make it difficult to figure out which mismatched HLA loci contribute mainly to the incidence and severity of GVHD, TRM and mortality, and which HLA locus mismatches has minimal impact on outcomes. Despite there are several guidelines published, evidence-based recommends have been absent so far [52–55]. Most recently, a published meta-analysis assess the impact of HLA-DPB1 allele mismatches on overall survival of patients receiving unrelated-donor HCT [56]. Other important end points were not mentioned, and many studies with large populations were not included in the review. Additionally, the analysis of individual HLA locus mismatches at the antigen level was not performed in the absence of sufficient data. As such, we undertake the meta-analysis in an effort to identify potential permissive HLA locus mismatches and candidate markers for protecting against primary disease relapse by means of systematically and comprehensively assessing the impacts of both individual HLA locus mismatches and number of HLA locus mismatches on multiple outcomes, which is of great benefit for ascertaining acceptable HLA minimal mismatched grafts and for predicting prognosis of patients after unrelated-donor HCT.

## RESULTS

### Study and patient characteristics

**Figure 1:**
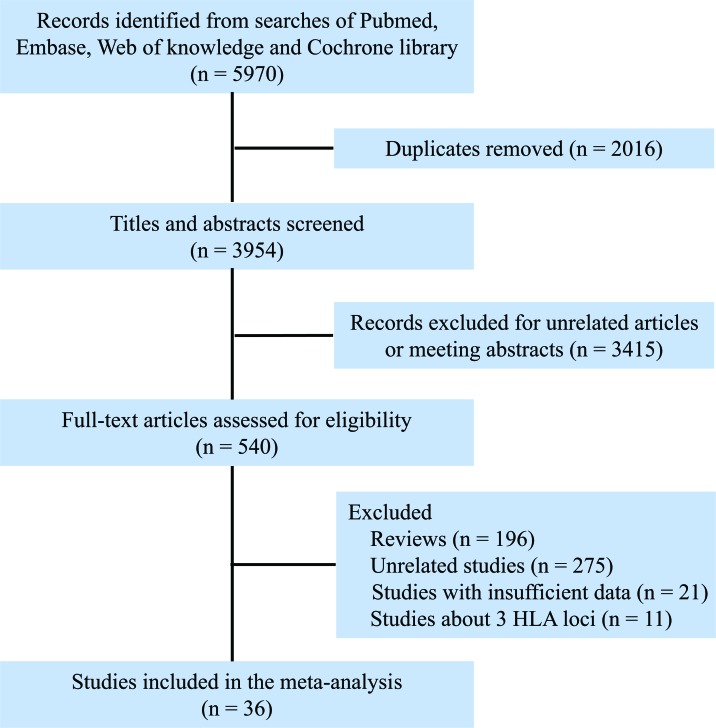
Flow chart for selection of studies

**Figure 2:**
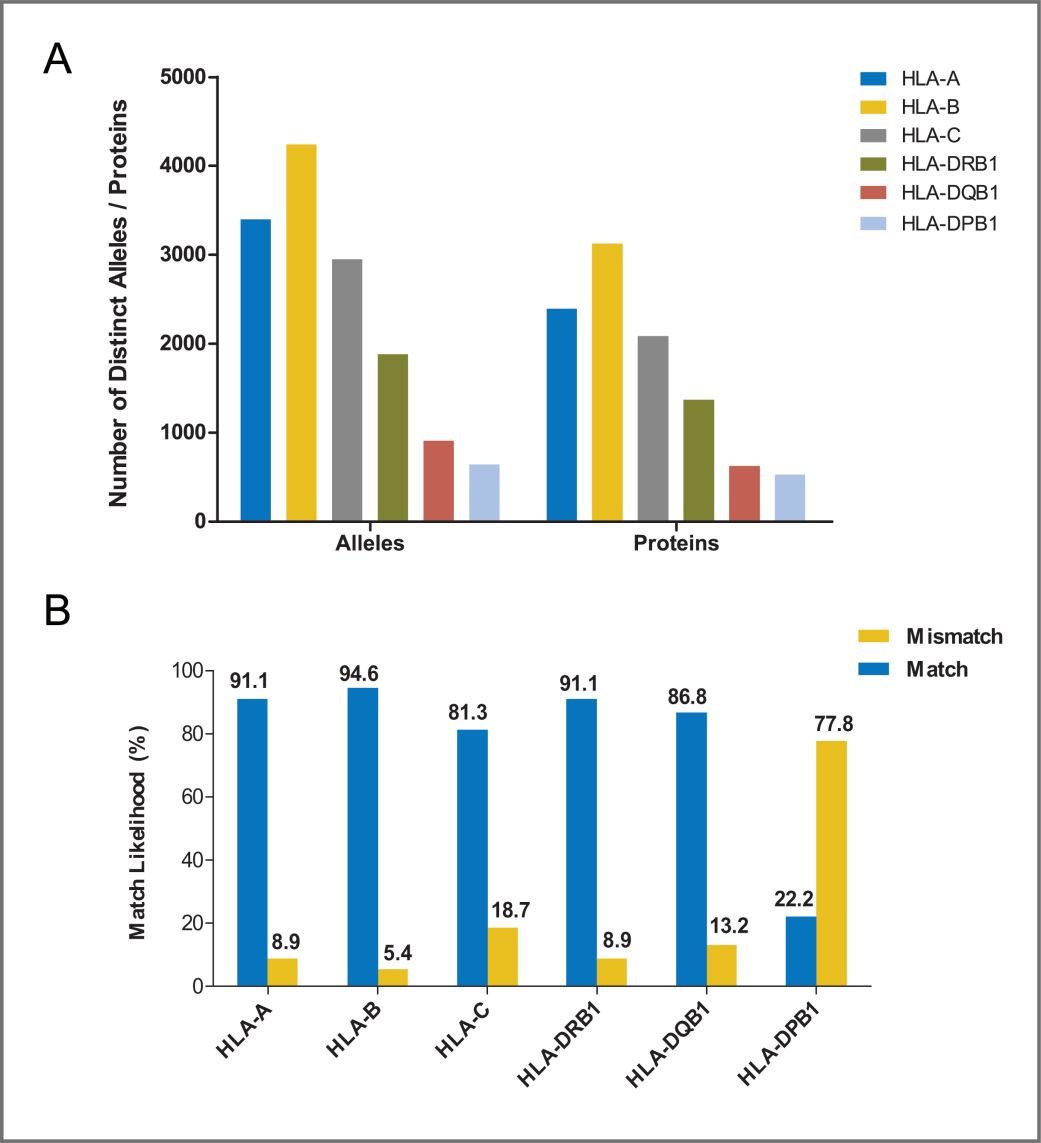
Polymorphism and match likelihood for individual HLA loci (A) allelic polymorphism and protein diversity for individual HLA loci, data were taken from http://www.ebi.ac.uk/imgt/hla/stats.html; accessed April, 2016. (B) match and mismatch likelihood of individual HLA loci.

The flow diagram of study search and selection was illustrated in Figure 1. The search strategy was showed in Supplementary Table 1. A total of 36 studies were included [16–51], of which, 15 studies analyzed 6 HLA loci [17, 18, 23, 24, 26, 28, 30, 31, 35, 42, 44–47, 50], 8 researches mentioned 5 HLA loci [16, 19–22, 27, 29, 38], and 14 studies investigated 4 HLA loci [25, 32–34, 37, 39–41, 43, 44, 48, 49, 51] (Table 1). Study characteristics were showed in Supplementary Table 2, all of the included studies were at low risk of bias. Polymorphism and matching likelihood at individual HLA loci were showed in Figure 2. Class I HLA alleles are more polymorphic than Class II HLA alleles, which was also seen in terms of protein diversity. The highest mismatch likelihood was seen in patients with HLA-DPB1 and -C locus mismatches.

**Table 1:** Patient, donor and transplantation characteristics according to number of HLA locus

Characteristic and stratum	Total	HLA 4 loci	HLA 5 loci	HLA 6 loci
**Number of studies**	36	14	8	15
**Patients, no. (%)**	100,072 (100)	47,837 (40.9)	10,932 (12.1)	41,303 (47.0)
**Patient age, median (range), y**	40.5 (0-81)	40.5 (0-81)	39.5 (0-79)	38.5 (0-77)
**Donor age, median (range), y**	41 (3-79)	39 (3-75)	48.5 (18-79)	35 (3-67)
**Disease at HCT, no. (%)**				
Acute lymphoblastic leukemia	22,210 (22.2)	10,543 (22.0)	1,907 (17.4)	9,760 (23.6)
Acute myeloblastic leukemia	37,115 (37.1)	21,996 (46.0)	2,681 (24.5)	12,438 (30.1)
Chronic myeloid leukemia	21,027 (21.0)	8,514 (17.8)	3,704 (33.9)	8,809 (21.3)
Myelodysplastic syndrome	10,654 (10.6)	6,010 (12.6)	994 (9.1)	3,650 (8.8)
Lymphoid malignancy	2,289 (2.3)	43 (0.1)	652 (6.0)	1,594 (3.9)
Aplastic anemia	881 (0.9)	275 (0.6)	112 (1.0)	494 (1.2)
Multiple myeloma	519 (0.5)	2 (<0.1)	280 (2.6)	237 (0.6)
Others	3,491 (3.5)	454 (0.9)	602 (5.5)	2,435 (5.9)
Missing	1,886 (1.9)	0	0	1,886 (4.6)
**Graft source, no. (%)**				
Bone marrow	69,941 (69.9)	26,909 (56.3)	7,305 (66.8)	35,727 (86.5)
Peripheral blood	29,797 (29.8)	20,928 (43.7)	3,483 (31.9)	5,386 (13.0)
Missing	334 (0.3)	0	144 (1.3)	190 (0.5)
**Disease status at HCT, no. (%)**				
Standard	41,857 (41.8)	24,523 (51.3)	3,572 (32.7)	13,762 (33.3)
High (intermediate and advanced)	47,092 (47.1)	21,906 (45.8)	6,647 (60.8)	18,539 (44.9)
Missing	11,123 (11.1)	1,408 (2.9)	713 (6.5)	9,002 (21.8)
**Performance status prior to HCT, no. (%)**				
<90	14,081 (14.0)	12,540 (26.2)	0	1,541 (3.7)
90-100	31,001 (31.0)	26,755 (55.9)	0	4,246 (10.3)
Missing	54,990 (55.0)	8,542 (17.9)	10,932 (100)	35,516 (86.0)
**Donor/recipient gender match, no. (%)**				
Male to male	30,547 (30.5)	12,462 (26.0)	3,990 (36.5)	14,095 (34.1)
Male to female	17,655 (17.6)	7,948 (16.6)	2,330 (21.3)	7,377 (17.9)
Female to male	14,715 (14.7)	5,839 (12.2)	1,809 (16.5)	7,067 (17.1)
Female to female	14,399 (14.4)	5,868 (12.3)	1,800 (16.5)	6,731 (16.3)
Missing	22,756 (22.7)	15,720 (32.9)	1,003 (9.2)	6,033 (14.6)
**Donor/recipient CMV serostatus, no. (%)**				
Negative/negative; −/−	15,474 (15.5)	11,192 (23.4)	105 (1.0)	4,177 (10.1)
Negative/positive; -/+	14,736 (14.7)	11,622 (24.3)	74 (0.7)	3,040 (7.4)
Positive/positive; +/+	9,265 (9.3)	6,726 (14.0)	68 (0.6)	2,471 (6.0)
Positive/negative; +/−	7,840 (7.8)	5,343 (11.2)	59 (0.5)	2,438 (5.9)
Missing	52,757 (52.7)	12,954 (27.1)	10,626 (97.2)	29,177 (70.6)
**Conditioning regimen, no. (%)**				
Myeloablative	76,240 (76.2)	38,240 (79.9)	8,248 (75.4)	29,752 (72.0)
Reduced intensity	13,423 (13.4)	8,977 (18.8)	1,321 (12.1)	3,125 (7.6)
Missing	10,409 (10.4)	620 (1.3)	1,363 (12.5)	8,426 (20.4)
**Total body irradiation, no. (%)**				
Yes	36,000 (36.0)	16,793 (35.1)	1,780 (16.3)	17,427 (42.2)
No	19,532 (19.5)	11,654 (24.4)	629 (5.7)	7,249 (17.6)
Missing	44,540 (44.5)	19,390 (40.5)	8,523 (78.0)	16,627 (40.2)
**GVHD prophylaxis, no. (%)**				
Cyclosporine based	29,584 (29.6)	15,675 (32.8)	2,986 (27.3)	10,923 (26.4)
Tacrolimus based	34,734 (34.7)	23,205 (48.5)	210 (1.9)	11,319 (27.4)
Cyclosporine or tacrolimus based	9,391 (9.4)	3,009 (6.3)	0	6,382 (15.5)
Others	5,314 (5.3)	2,505 (5.2)	293 (2.7)	2,516 (6.1)
Missing	21,049 (21.0)	3,443 (7.2)	7,443 (68.1)	10,163 (24.6)
**T-cell depletion, no. (%)**				
Yes	18,001 (18.0)	9,781 (20.4)	1,669 (15.3)	6,551 (15.9)
No	67,387 (67.3)	26,258 (54.9)	8,864 (81.1)	32,265 (78.1)
Missing	14,684 (14.7)	11,798 (24.7)	399 (3.6)	2,487 (6.0)
**Year of transplantation**				
	1988-2012	1988-2011	1988-2010	1988-2012

Abbreviations: HLA 4 Loci, HLA-A, -B, -C, and -DRB1 loci; HLA 5 Loci, HLA-A, -B, -C, DRB1 and -DQB1 loci; HLA 6 Loci, HLA-A, -B, -C, DRB1,-DQB1 and -DPB1 loci; HCT, hematopoietic cell transplantation; CMV, cytomegalovirus.

### Acute GVHD

**Figure 3:**
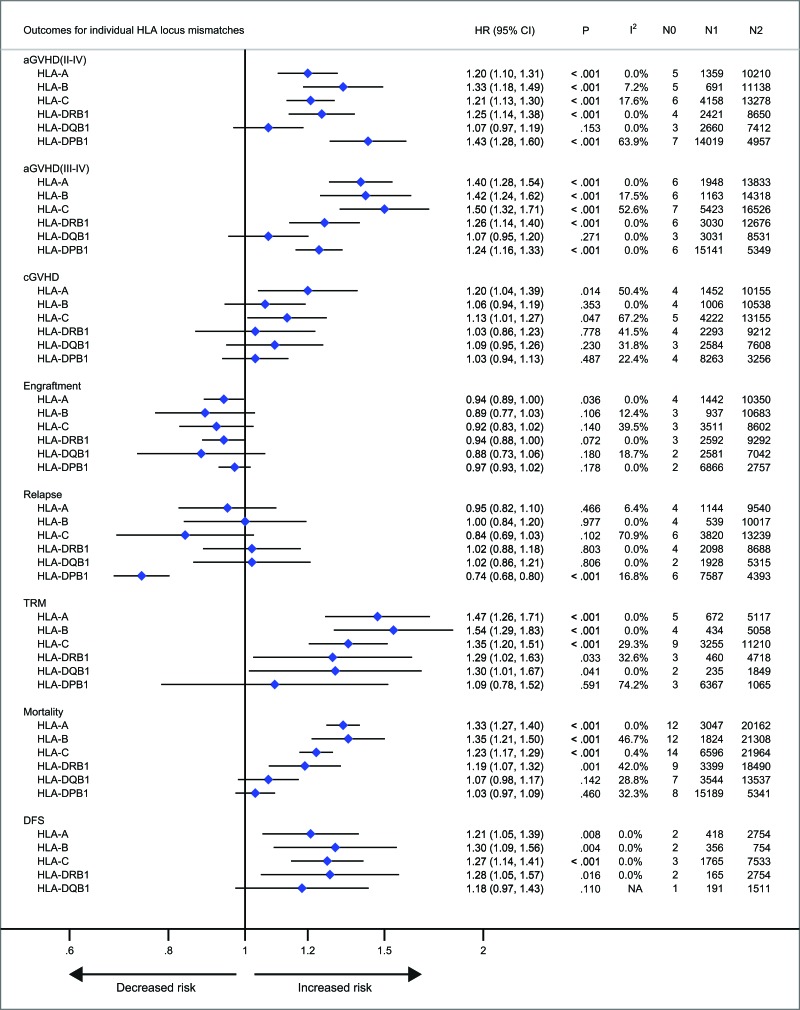
Individual HLA locus mismatches versus corresponding controls Pooled hazard ratios (HRs) and 95% CIs for post-transplantation end points. N0, number of studies; N1, number of patients with a specific HLA locus mismatches; N2, number of patients as corresponding controls; NA, not available.

**Figure 4:**
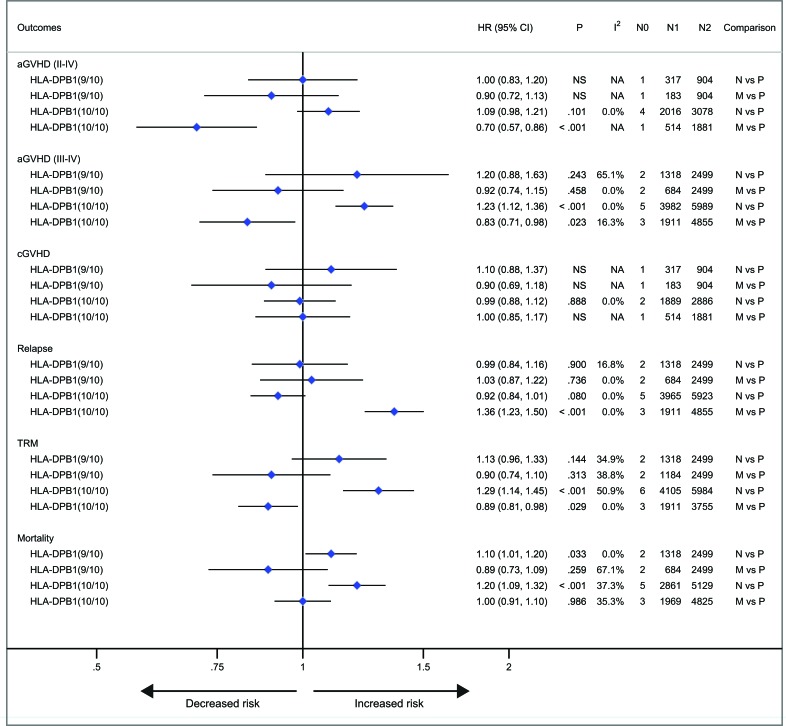
Nonpermissive mismatched or matched HLA-DPB1 alleles versus permissive mismatched HLA-DPB1 alleles Pooled hazard ratios (HRs) and 95% CIs for post-transplantation end points. N0, number of studies; N1, number of patients as the case; N2, number of patients as the control. 9/10, comparisons in the population with 9/10 HLA matching; 10/10, comparisons in the population with 10/10 HLA matching. N vs P, nonpermissive mismatch versus permissive mismatch; M vs P, match versus permissive mismatch. NS, not significant; NA, not available.

**Figure 5:**
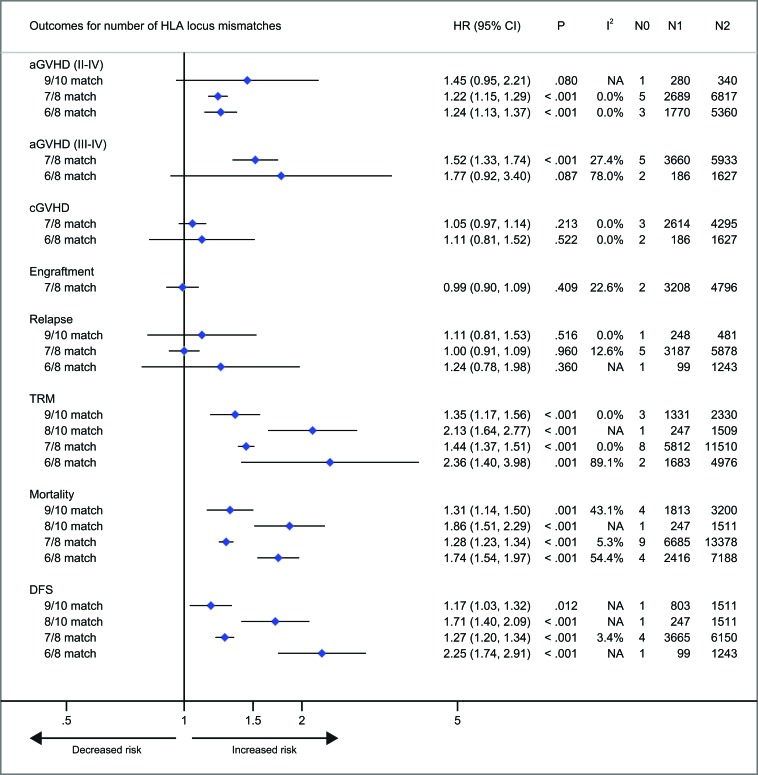
Number of HLA locus mismatches Pooled hazard ratios (HRs) and 95% CIs for post-transplantation end points. 7/8 or 6/8 HLA matching versus 8/8 HLA matching; 9/10 or 8/10 HLA matching versus 10/10 HLA matching. N0, number of studies; N1, number of patients as the case; N2, number of patients as the control; NA, not available.

With respect to recipients with HLA-A, -B, -C, -DRB1, or -DPB1 locus mismatches, the risk of acute GVHD (III-IV) was significantly higher, with hazard ratios of 1.40 (95% CI, 1.28 to 1.54; *P* < .001), 1.42 (95% CI, 1.24 to 1.62; *P* < .001), 1.50 (95% CI, 1.33 to 1.69; *P* < .001; I^2^ = 58.5%), 1.26 (95% CI, 1.14 to 1.40; *P* < .001) and 1.24 (95% CI, 1.16 to 1.33), respectively, as compared to controls (Figure 3). However, HLA-DQB1 mismatches did not have a significant impact on acute GVHD (III-IV) (HR, 1.07; 95% CI, 0.95 to 1.20; *P* = .271) (Figure 3). The effect of individual HLA mismatches was replicated for acute GVHD (II-IV), with substantial heterogeneity in the analysis of HLA-DPB1 locus (I^2^ = 63.9%) (Figure 3). Secondly, we investigated the impact of nonpermissive HLA-DPB1 mismatches on aGVHD (Figure 4). Among patients with 10/10 HLA matching, nonpermissive HLA-DPB1 mismatches were associated with a significantly increased risk of acute GVHD (III-IV) (*P* < .001), and had a trend of slight increasing risk of acute GVHD (II-IV) (*P* = .101). Conversely, matched HLA-DPB1 was significantly associated with decreased incidence of acute GVHD (II-IV) (*P* < .001) and GVHD (III-IV) (*P* = .023). In the 9/10 HLA matching population, both nonpermissive mismatched and matched HLA-DPB1 did not have statistically significant impacts on grade II-IV or III-IV acute GVHD (all *P* > .05). Thirdly, we assessed the risks of aGVHD for number of HLA locus mismatches (Figure 5). Compared with recipients with 8/8 HLA matching, those with 7/8 HLA matching had a higher risk of acute GVHD (II-IV) (*P* < .001) and GVHD (III-IV) (*P* < .001); those with 6/8 matches had a higher risk of acute GVHD (II-IV) (*P* < .001), and had a trend of increased incidence of acute GVHD (III-IV) (*P* = .087; I^2^ = 78.0%). Only one study assessed the risk of the acute GVHD (II-IV) for 9/10 HLA matches, compared with 10/10 matches (*P* = .080).

### Chronic GVHD

Patients with HLA-A locus mismatches had a higher risk of chronic GVHD (HR, 1.20; 95% CI, 1.04 to 1.39; *P* = .014; I^2^ = 50.4%), compared with the control (Figure 3). Similarly, HLA-C locus mismatches slightly increased hazard of chronic GVHD with a borderline significance (HR, 1.13; 95% CI, 1.01 to 1.27; *P* = .047; I^2^ = 67.2%) (Figure 3). However, mismatches at other HLA loci had no significant impact on chronic GVHD (all *P* > .05) (Figure 3). Secondly, both nonpermissive mismatched and matched HLA-DPB1 had no impact on the incidence of chronic GVHD (all *P* > .05) (Figure 3). Thirdly, compared with 8/8 HLA matches, neither 7/8 nor 6/8 matches showed a higher risk of chronic GVHD (*P* = .213 and .522, respectively).

### Neutrophil engraftment

As shown in Figure 3, there was a trend of promoting neutrophil engraftment for recipients with individual HLA locus mismatches. Recipients with 7/8 HLA matching did not have an impact on neutrophil engfratment, compared with 8/8 matching (Figure 5).

### Relapse

Mismatches at HLA-DPB1 locus was significantly associated with a decreased risk of primary disease relapse, compared with the control (HR, 0.74; 95% CI, 0.68 to 0.80; *P* < .001) (Figure 3). HLA-C locus mismatches has a trend of decreased relapse (HR, 0.84; 95% CI, 0.69 to 1.03; *P* = .102; I^2^ = 70.9%) (Figure 3). Mismatches at HLA-A, -B, -DRB1 or DQB1 locus had no significant impact on disease relapse (all *P* > .05) (Figure 3). Secondly, in the population with 10/10 HLA matching, nonpermissive HLA-DPB1 mismatches had a trend of reduced disease relapse, (*P* = .080) (Figure 4), whereas matched HLA-DPB1 had a higher risk of relapse (*P* < .001) (Figure 4). That the impact of nonpermissive mismatched and matched HLA-DPB1 on disease relapse was not observed among the patients with 9/10 HLA matching (*P* = .900 and *P* = 0.736) (Figure 4). Thirdly, the impact on relapse was not observed in patients with 9/10, 7/8 and 6/8 HLA matching (*P* = .516, .960 and .360, respectively) (Figure 5).

### TRM, mortality and DFS

For recipients with mismatches at HLA-A, -B, -C -DRB1 or -DQB1 locus, the risk of TRM was significantly higher, as compared to controls, with hazard ratios of 1.47 (95% CI, 1.26 to 1.71; *P* < .001), 1.54 (95% CI, 1.29 to 1.83; *P* < .001), 1.35 (95% CI, 1.20 to 1.51; *P* < .001), 1.29 (95% CI, 1.02 to 1.63; *P* = .033) and 1.30 (95% CI, 1.01 to 1.67; *P* = .041), respectively (Figure 3). Whereas, HLA-DPB1 mismatches had no significant impact on TRM (*P* = .591) (Figure 3). With respect to mortality, intriguingly, similar results were observed for HLA-A, -B, -C, -DRB1 or DPB1 mismatches, with hazard ratios of 1.33 (95% CI, 1.27 to 1.40; *P* < .001), 1.35 (95% CI, 1.21 to 1.50; *P* < .001), 1.23 (95% CI, 1.17 to 1.29; *P* < .001), 1.19 (95% CI, 1.07 to 1.32; *P* = .033) and 1.03 (95% CI, 0.97 to 1.09; *P* = .460), respectively (Figure 3). Whereas HLA-DQB1 locus mismatches had no significant impact on mortality (*P* = .460), which is inconsistent with it for TRM (Figure 3). These data demonstrated that the pooled point estimates of class I HLA loci were prone to be greater than those of class II HLA loci, with respect to TRM and mortality. Additionally, the effect of mismatches at HLA-A, -B, -C, -DRB1 or -DQB1 locus was replicated for DFS, with few studies investigating this end point (Figure 3).

Secondly, we investigated the impact of nonpermissive HLA-DPB1 mismatches on TRM and mortality (Figure 4). In the 10/10 HLA matching population, the risk of TRM and mortality was significantly greater for nonpermissive HLA-DPB1 mismatches (*P* < .001 and *P* < .001, respectively). In contrast, matched HLA-DPB1 had a marginally significant effect of protecting against TRM (*P* = .029). But for mortality, the impact of matched HLA-DPB1 was not identified (*P* = .986). Among the 9/10 HLA matching patients, matched HLA-DPB1 did not result in a decreased risk of TRM and mortality (*P* = .313 and *P* = .259, respectively); similarly, the impact on TRM was replicated in patients with nonpermissive HLA-DPB1 mismatches (*P* = .144), but increasing risk of mortality was observed for the mismatches, with a borderline significance (HR, 1.10; 95% CI, 1.01 to 1.20; *P* = .033).

Thirdly, as shown in Figure 5, compared with 10/10 matching, there was a significantly increased risk of TRM and mortality for both 9/10 and 8/10 HLA mismatches. Furthermore, the pooled point estimate of 8/10 HLA mismatches was greater than it of 9/10 mismatches. The findings were replicated in both 7/8 and 6/8 HLA mismatches, compared with 8/8 HLA matching. And similar results were observed in terms of DFS.

### Stratified analyses

Stratified analysis was showed in STables S3-5 according to combinations of HLA allele or antigen mismatches. Only one study analyzed 1 or 2 antigen mismatches so that the pooled analysis could not be performed [38].

## DISCUSSION

### Summary main results

We found HLA-DQB1 locus mismatches had no significant impact on multiple outcomes except for TRM, it is a potential candidate of permissive HLA locus mismatches. Secondly, we attempted to identify several candidates serving as remarkable graft-*versus*-tumor effects (GVT). HLA-DPB1 locus mismatches had a significantly protective effect against leukemia relapse, which was attributed to nonpermissive HLA-DPB1 mismatches. Meanwhile, mismatched HLA-DPB1 had no significant impact on chronic GVHD, TRM or mortality. But relative to permissive mismatches, nonpermissive HLA-DPB1 mismatches had a significantly increased risk of TRM and mortality in 10/10 HLA matching. Similarly, HLA-C locus mismatches had a trend of reduced risk of relapse, but had a significant increased risk of TRM and mortality and a slightly increased risk of chronic GVHD. Thirdly, mismatches at HLA-A, -B, -DRB1 loci significantly increased the risks of acute GVHD, TRM and mortality, but had no significant protection against primary disease relapse.

### Agreements and disagreements with other studies

Most recently, one meta-analysis demonstrated that 9/10 HLA matching had a higher risk of mortality, compared with 10/10 matching, with hazard ratio of 1.27 (95% CI, 1.12 to 1.45; *P* < .001) [56], which was similar to ours (HR, 1.31; 95% CI, 1.14 to 1.50; *P* = .001). In addition, the risk for individual HLA allele mismatches was similar with it in our stratified analyses. However, multiple comparisons for other important outcomes were not performed in the pool-analysis. Many low-quality studies with small sample size were included, which did not meet our eligible criteria. To our knowledge, our meta-analysis is the first to systematically and comprehensively assess the impact of HLA locus mismatches on clinical outcomes in unrelated donor HCT.

### Strengths and limitations of this study

Our meta-analysis had several strengths. Firstly, a large number of patients were included to obtain a bigger statistical power for a given comparison. Secondly, a series of end points were assessed in an effort to obtain a comprehensive recognition about the effect of individual HLA locus mismatches. Thirdly, the risk of individual HLA locus mismatches was similar among analogous end points, which contributed to the robustness of pooled estimates. For instance, mismatches at HLA-DQB1 locus had no impact on both acute GVHD (II-IV) and acute GVHD (III-IV). Furthermore, HLA-DQB1 locus mismatches were better tolerated than other HLA loci for acute GVHD. In terms of TRM, the pooled point estimates of class I HLA molecules were greater than those of class II HLA molecules, as observed for mortality. Fourthly, given the highest mismatched likelihood of HLA-DPB1 alleles, we explored the impact of nonpermissive HLA-DPB1 locus mismatches on multiple end points. Fifthly, we assessed the impact of number mismatches of HLA loci on outcomes. Sixthly, Donor-recipient baseline characteristics were summarized together in order to demonstrate the practical application field of pooled results.

There were several limitations in our meta-analysis. First of all, all of our main pooled estimates belonged to average effects for individual HLA loci, with combining results from different studies presented separately as 1 allele mismatching, 1 or 2 allele mismatching or a single mismatching. Few studies investigated the effect of 2 antigen mismatches at individual HLA loci [38]. Second, clinical heterogeneity from individual studies such as donor age, patients’ performance status, primary disease, disease status at HCT, intensity of conditioning regimen and GVHD prophylaxis, grafts with T cell depletion, were difficult to be completely balanced between cases and controls, especially in studies with relatively small sample size [17, 20, 22, 32, 45]. Third, bone marrow transplantation reduced the risk of chronic GVHD but increase the risk of graft failure, compared with peripheral blood transplantation [57, 58]. In our study, 69.9% patients received bone marrow derived hematopoietic cells, which might attenuate the risk of individual HLA locus mismatches for chronic GVHD and neutrophil engraftment. Similarly, the inclusion of anti-thymocyte globulin (ATG) into conditioning regimen for patients with leukemia resulted in significantly decreased risk of chronic GVHD after allogeneic transplantation [59, 60], but the effect of HLA locus mismatching was not analyzed according to the application of ATG in included studies. Fourth, with respect to the same primary disease, the therapy strategy has been evolving over time, which might decrease the incidence of complications [17, 61]. Fifth, HLA genes match likelihood at high resolution varied across different race and ethnicity groups [62], detailed information was not presented in many studies. Sixth, HLA locus mismatching had a higher risk of mortality in recipients with standard-risk disease compared those with high-risk disease [19, 27]. In our meta-analysis, less than half of the population was standard-risk disease status at HCT. Seventh, significant heterogeneity mainly existed in the mismatches at the HLA-A, -C and -DPB1 loci. We conducted a sensitivity analysis to figure out the robustness of pooled results. As shown in Supplementary Figure S1, using the trim and fill method, the robustness of all of the pooled estimates were presented in both fixed and random-effects models [55]. It is notable that HLA-C locus mismatches had a significantly reduced risk of relapse compared with matched control (*P* < .001 and *P* = .017 respectively). We stratified the HLA-C locus mismatches into three groups: 1 allele, 1 antigen and 1 or 2 allele mismatches, the hazard ratios of relapse were 0.88 (95% CI, 0.70 to 1.10; *P* = .259; I^2^ = 33.7%), 1.04 (95% CI, 0.91 to 1.20; *P* = .531; I^2^ = 0.0%) and 0.70 (95% CI, 0.62 to 0.79; *P* < .001; I^2^ = 0.0%), respectively. The heterogeneity appeared to be found, but results of the subgroups were less robust for fewer studies used in each pooled analysis. To explore the heterogeneity of HLA-DPB1 mismatches for acute GVHD (II-IV), we excluded 2 studies with small sample size and 1 study with GVH direction mismatches, and then pooled the remaining results, with hazard ratio of 1.34 (95% CI, 1.27 to 1.42; *P* < .001; I^2^ = 0.0%), which was consistent with it from the primary analysis. We failed to reveal possible sources of heterogeneity for TRM at HLA-DPB1 locus, and for chronic GVHD and acute GVHD (III-IV) at HLA-C locus. Eighth, we were unable to assess publication bias because of relatively few studies for most of the end points. Ninth, specific HLA genotype mismatching combination among donor-recipient pairs was investigated in few studies, the pool-analysis could not be performed [31, 43]. Last but not least, in the absence of more detailed information, predefined subgroup analyses could not be conducted.

### Assessment of HLA locus mismatches in terms of expression levels or amino acid substitutions

Most recent studies attempted to identify permissive mismatches in terms of expression levels of HLA-C and -DPB1 [63, 64]. With respect to HLA-C molecule, patients with higher expression of mismatched HLA-C tended to suffer from higher risk of actue GVHD (III-IV) and nonrelapse mortality compared with those with lower expression mismatches. Furthermore, the definitive correlation of most HLA-C allotypes with their corresponding expression levels will be beneficial to the selection of permissive mismatched donors in terms of HLA genotype. A similar finding was identified in HLA-DPB1 mismatches. When donors with low-expression HLA-DPB1 molecules, recipients with mismatched high-expression HLA-DPB1 had a higher risk of acute GVHD (II-IV), compared with patients with low-expression HLA-DPB1 mismatches. It is found that high-expression HLA-DPB1 correlated with its single- nucleotide variant (rs9277534G) of the sixth exon in the 3’ untranslated region. In contrast, HLA-DPB1 expression was lower when with the rs9277534A variant. A possible explanation is that non-coding RNA might mediate the HLA-DPB1 RNA silencing through binding rs9277534A. In addition, some studies attempted to identify specific nonpermissive HLA locus mismatches according to amino acid substitutions (AAS) at key peptide-binding residues of HLA molecules. For example, among the population with a single HLA-C mismatches, ASS at position 116 had a significantly increased risk of acute GVHD (III-IV), compared with those without the AAS [65].

## MATERIALS AND METHODS

This meta-analysis is reported according to the PRISMA statement [66]

### Eligibility criteria

Studies should be included when meeting the following criteria: (1) patients receiving unrelated-donor HCT; (2) patients with hematological disorders; (3) high-resolution typing was performed as described for HLA-A, -B, -C, -DRB1, -DQB1, and/or -DPB1 loci; (4) investigating the impact of HLA locus mismatching on clinical outcomes; (5) cohort studies. If a study meet any of the following criteria, it should be excluded: (1) HLA locus mismatch combinations; (2) analysis of HLA protein expression; (3) mismatched HLA alleles as controls except for HLA-DPB1; (4) data presented as percentage; (5) unrelated *versus* related donor HCT; (6) meeting abstract or case report.

### Study searching and selection

We searched four databases (PubMed, Embase, Web of Science and the Cochrane Library) from inception to February 2016, with these keyword combinations involving “hematopoietic”, “hematologic” or “transplantation”; “unrelated”; “human leukocyte antigen” or “major histocompatibility complex”; and “mismatch” or “mismatched”. The complete search strategy is available in the appendix (Supplementary Table 1). Two reviewers (R.T. and T.Z.) independently selected studies based on the eligibility criteria. Disagreements were resolved through discussing with a third reviewer (B.Y.).

### Definition of end points

Primary end points included grade II to IV aGVHD, grade III to IV aGVHD, chronic GVHD (cGVHD), neutrophil engraftment and disease relapse. The incidence of grades II-IV or III-IV acute GVHD was defined according to the Glucksberg scale [67]. Chronic GVHD included limited and extensive conditions and was defined according to the Seattle criteria [68]. Neutrophil engraftment was defined achieving an absolute neutrophil count > 0.5×10^9^/L for 3 consecutive days after transplantation. Relapse was regarded as recurrence of primary leukemia or myelodysplastic syndrome (MDS). Secondary end points were as follows: transplant-related mortality (TRM), Mortality and disease-free survival (DFS). Overall mortality was defined as time from HCT to death from any cause. TRM was death without evidence of primary disease recurrence after HCT. DFS was defined as time to relapse of primary disease or death from any cause.

### Risk of bias within and across studies

Risk of bias within studies was assessed independently by the two authors (R.T. and T.Z.) using the Newcastle-Ottawa Scale (NOS) components for cohort studies [69]. Studies were scored to be low risk of bias (≥ 3 points) or higher risk of bias ( < 3 points). Disagreement was resolved through discussing with a third author (B.Y.). Publication bias was not investigated, because of less than 10 studies included for most of the end points.

### HLA typing

In our meta-analysis, high-resolution HLA typing refers to obtaining diversity of the allele sequence at individual HLA loci for donor-recipient pairs, using various methods such as sequencing based typing [70], sequence specific priming [71], reference strand conformation analysis [72], sequence specific oligonucleotide probing [73] and so on. Low-resolution HLA (antigen or serologic level) disparities are derived from converting high-resolution typing to its corresponding serologic equivalents, except for a few HLA-B alleles mapping to their specific equivalents [74].

### HLA matching

Whenever assessing the effect of individual HLA locus mismatches, we predefined controls as patients with corresponding HLA allele matching adjusted with other HLA allele matching, or those with complete HLA allele matching. Individual HLA locus mismatches involved allele-level (1 or 2 alleles), antigen-level (1 or 2 antigens) and/or a single (1 allele or 1 antigen) mismatches, as presented in studies. Secondly, there were at most 6 HLA loci (HLA-A, HLA-B, HLA-C, HLA-DRB1, HLA-DQB1 and HLA-DPB1) investigated in some studies. High-resolution matching at 5 loci (except for HLA-DPB1) is designated as 10/10 HLA matching, HLA 9/10 matching refers to donor-recipient pairs with a single mismatches at any one of the 5 loci, HLA 8/10 matching includes two allele or antigen mismatches at one or two of the 5 HLA loci; and high-resolution matching at 4 loci (except for HLA-DPB1 and HLA-DQB1) is designated as 8/8 HLA matching, HLA 7/8 matching was defined as donor-recipient pairs with a single mismatches at any one of the 4 loci, HLA 6/8 matching includes two mismatch (allele or antigen) at one or two of the 4 HLA loci. Additionally, in a population with HLA-DPB1 allele mismatches, permissive HLA-DPB1 mismatches are T-cell-epitope group matches, whereas nonpermissive HLA-DPB1 mismatches belong to T-cell-epitope group mismatches in either graft-*versus*-host or host-*versus*-graft direction [30, 35, 42].

### Data extraction

Data were extracted as follows: (1) the baseline characteristics of donor-recipient pairs and individual studies; (2) data presented as multivariate-adjusted point estimates and corresponding 95% CIs for each comparison. Among the included studies, some data were showed as HR, others were presented as relative risk (RR) or odds ratio (OR). We uniformed effect measure as HR in our meta-analysis. Two reviewers (R.T. and T.Z.) independently extracted these data using a spreadsheet developed specifically for the meta-analysis. Discrepancies were resolved through discussing with a third reviewer (B.Y.).

### Data synthesis and analysis

Primary analyses compared mismatches at individual HLA loci with corresponding controls. Separately, we assessed the impact of nonpermissive mismatches and matches at HLA-DPB1 locus on multiple end points, as compared to permissive HLA-DPB1 mismatches. Secondary analyses evaluated the impact of number of HLA locus mismatches on multiple end points. In addition, we summarized the baseline characteristics of included studies and patients separately, and calculated the matching likelihood of individual HLA loci based on a larger population. We pooled HRs and 95% CIs using the Mantel-Haneszel random-effects model for each comparison [75, 76]. The magnitude of between-studies heterogeneity was assessed using the I^2^ statistic, with value ≥ 50% indicating substantial heterogeneity, I^2^ value < 50% was not showed in the text [77–79].

### Sensitivity and subgroup analyses

Firstly, with respect to pooled estimates with a substantial heterogeneity, we performed a sensitivity analysis using the trim and fill adjustment method (random and fixed effects linear estimator) in an effort to investigate the robustness of primary synthesized results [80]. Secondly, we did stratified analysis according to mismatched level (allele and/or antigen) at individual HLA loci. Additional subgroups analyses should be performed according to the quality of studies, direction of HLA locus mismatching, patient or donor age, disease status before HCT, intensity of conditioning regimen, grafts with T-cell depletion, GVHD treatment, cytomegalovirus serostatus of donor-recipient pairs, duration of follow up.

All statistical tests were 2-sided, *P* value < .05 was considered statistically significant. The meta-analysis was performed using STATA/SE version 12.0.

## CONCLUSIONS

We identify HLA-DQB1 locus mismatches as a permissive mismatching, which offers HCT choices for patients without all 5 HLA-locus matched grafts. HLA-DPB1 locus nonpermissive mismatches have a significantly protective effect against leukemia relapse, simultaneously have no significantly increased risk of TRM, mortality or DFS. HLA-C locus mismatches have a trend of protecting against leukemia relapse. Further researches should be conducted to confirm our findings using individual patient data meta-analysis, and should assess the impacts of individual HLA locus mismatches on multiple outcomes in terms of the allele and antigen levels respectively. Subgroup analysis should be performed according to disease category, disease status at HCT, intensity of conditioning regimen, T-cell depletion or not, and so on. More studies are needed to identify and verify permissive mismatches at HLA-C and DPB1 loci in unrelated donor HCT.

## SUPPLEMENTARY MATERIALS TABLES


