# Supplementary material for: Clinical implications of HLA locus mismatching in unrelated donor hematopoietic cell transplantation: a meta-analysis
Source: Oncotarget. 2017 Feb 11;8(16):27645–60. doi: 10.18632/oncotarget.15291 (PMC5432365; doi:10.18632/oncotarget.15291)
Supplement: Supplementary file 1 [file oncotarget-08-27645-s001.docx]

**Table S1: Search strategy.**

| **Database** | **History** | **Items found** |
| --- | --- | --- |
| Pubmed | ((unrelated[Tittl/Abstract]) AND ((((human leukocyte antigen) OR HLA) OR major histocompatibility complex) OR MHC)) AND ((transplantation[Title/Abstract]) AND (((hematopoietic[Title/Abstract] OR haematopoietic[Title/Abstract]) OR hematologic[Title/Abstract])). | 1507 |
| Embase | #1 ‘hematopoietic’: ab,ti; #2 ‘haematopoietic’: ab,ti; #3 ‘hematologic’: ab,ti; #4 ‘transplantation’: ab,ti; #5 ‘human leukocyte antigen’: ab,ti; #6 ‘HLA’: ab,ti; #7 ‘major histocompatibility complex’: ab,ti; #8 ‘mhc’: ab,ti; #9 ‘unrelated’: ab,ti; #10 ‘mismatch’: ab,ti; #11 ‘mismatched’: ab,ti;  #12: #1 OR #2 OR #3; #13: #5 OR #6 OR #7 OR #8; #14: #10 OR #11; #15: #4 AND #12 AND #13 AND #14. | 2335 |
| Web of science | #1 TOPIC: (hematopoietic) OR TOPIC: (haematopoietic) OR TOPIC: (hematologic);  #2 TOPIC: (human leukocyte antigen) OR TOPIC: (HLA) OR TOPIC: (major histocompatibility complex) OR TOPIC: (MHC); #3 TOPIC: (unrelated); #4 TOPIC: (transplantation); #5 TOPIC: (mismatched) OR TOPIC: (mismatch);  #6: #1 AND #2 AND #3 AND #4 AND #5. | 2053 |
| The Cochron library | #1“major histocompatibility complex”: ab,ti,kw or “MHC”: ab,ti,kw or “human leukocyte antigen”: ab,ti,kw or “HLA”: ab,ti,kw; #2hematopoietic: ab,ti,kw or haematopoietic: ab,ti,kw or hematologic: ab,ti,kw; #3 unrelated: ab,ti,kw;  #4: #1 AND #2AND #3. | 75 |

**Table S2: Characteristics of included studies.**

| **First author (Year)** | **Journal** | **Region** | **Year of HCT** | **High-resolution**  **HLA typing** | **HLA loci used**  **for analysis** | **No. of patients** | | **Statistics** | **NOS**  **scores** |
| --- | --- | --- | --- | --- | --- | --- | --- | --- | --- |
| Elisabetta Zino （2004） | Blood | Europe | 1995-2002 | PCR-SBT, PCR-SSP, PCR-SSOP, RSCA | HLA-A, -B, -C, -DRB1, -DQB1 and -DPB1 | 118 | HR | | 5 |
| Neal Flomenberg (2004) | Blood | USA | 1988-1996 | PCR-SBT, PCR-SSOP | HLA-A, -B, -C, -DRB1, -DQA1, -DQB1, -DPA1 and -DPB1 | 1,874 | RR and OR | | 8 |
| Bronwen E.Shaw (2006) | Blood | Europe | 1996-2003 | Not available | HLA-A, -B, -C, -DRB1, -DQB1 and -DPB1 | 423 | HR and OR | | 6 |
| Bronwen E.Shaw (2007) | Blood | USA | 1984-2005 | PCR-SBT, PCR-SSP, PCR-SSOP, RSCA | HLA-A, -B, -C, -DRB1, -DQB1 and -DPB1 | 5,929 | HR | | 9 |
| Yasuo Morisbima (2007) | BBMT | Japan | 1993-2000 | PCR-SSP, Luminex  microbead method | HLA-A, -B, -C, -DRB1, -DQB1 and -DPB1 | 1,790 | HR | | 7 |
| Katarina Ludajic (2008) | BJH | Europe | 1995-2007 | PCR-SBT,  PCR-SSP | HLA-A, -B, -C, -DRB1, -DQB1 and -DPB1 | 161 | HR | | 6 |
| Roberto Crocchiolo (2009) | Blood | Europe | 1999-2006 | PCR-SBT, PCR-SSP, PCR-SSOP | HLA-A, -B, -C, -DRB1, -DQB1 and -DPB1 | 621 | HR | | 7 |
| Takakazu Kawase (2009) | Blood | Japan | 1993-2005 | PCR-SBT | HLA-A, -B, -C, -DRB1, -DQB1 and -DPB1 | 4,643 | HR | | 8 |
| Katharina Fleischhauer (2012) | Lancet Oncology | Japan  Non-Japan | 1993-2007 | PCR-SBT, PCR-SSP, PCR-SSOP, RSCA | HLA-A, -B, -C, -DRB1, -DQB1 and -DPB1 | 8,539 | HR and OR | | 9 |
| Joseph Pidala (2014) | Blood | Europe  Canada | 1999-2011 | PCR-SSP, PCR-SSOP | HLA-A, -B, -C, -DRB1, -DQB1 and -DPB1 | 5,015 | RR | | 9 |
| K Fleischhauer （2014） | BMT | USA | 1988-2000 | PCR-SSP, PCR-SSOP | HLA-A, -B, -C, -DRB1, -DQB1 and -DPB1 | 1,281 | HR | | 8 |
| Kirsten A.Thus (2014) | BBMT | Europe | 2007-2012 | PCR-SBT | HLA-A, -B, -C, -DRB1, -DQB1 and -DPB1 | 80 | HR | | 5 |
| K Gagne (2015) | BMT | Europe | 2000-2008 | PCR-SSP,  PCR-SBT | HLA-A, -B, -C, -DRB1, -DQB1 and -DPB1 | 1,342 | RR | | 9 |
| Yasuo Morishima (2015) | Blood | Japan | 1993-2010 | PCR-SBT, PCR-SSOP | HLA-A, -B, -C, -DRB1, -DQB1 and -DPB1 | 7,898 | RR | | 9 |
| Aiko Sato-Otsubo MS (2016) | Blood | Japan | 1993-2005 | PCR-SSP, Luminex  microbead method | HLA-A, -B, -C, -DRB1, -DQB1 and -DPB1 | 1,589 | HR | | 8 |
| Yasuo Morishima (2002) | Blood | Japan | 1993-1998 | PCR-SSP,  PCR-SBT | HLA-A, -B, -C, -DRB1 and -DQB1 | 1,298 | HR | | 6 |
| Effie W. Petersdorf （2004） | Blood | USA | 1985-2003 | PCR-SBT | HLA-A, -B, -C, -DRB1 and -DQB1 | 948 | HR | | 7 |
| J-M Tiercy (2004) | BMT | Europe | 1988-1999 | PCR-SSP, PCR-SSOP | HLA-A, -B, -C, -DRB1, and -DQB1 | 114 | RR | | 5 |
| VT Ho (2006) | BMT | USA | 2000-2004 | PCR-SSP,  RFLP | HLA-A, -B, -C, -DRB1 and -DQB1 | 111 | HR | | 5 |
| Y Chalandon (2006) | BMT | Europe | 1990-2004 | PCR-SSP, PCR-SSOP | HLA-A, -B, -C, -DRB1, and -DQB1 | 214 | RR | | 5 |
| E.W.Petersdorf (2007) | Tissue Antigens | Japan  Europe | Not available | PCR-SSP,  RSCA | HLA-A, -B, -C, -DRB1 and -DQB1 | 4,796 | HR | | 8 |
| R Crocchiolo (2009) | BMT | Europe | 1999-2006 | Not available | HLA-A, -B, -C, -DRB1 and -DQB1 | 805 | HR | | 6 |
| Daniel F¨urst (2013) | Blood | Europe | 1997-2010 | PCR-SBT, PCR-SSP, PCR-SSOP | HLA-A, -B, -C, -DRB1 and -DQB1 | 2,646 | HR | | 9 |
| Stephanie J. Lee (2007) | Blood | USA | 1988-2003 | PCR-SBT, PCR-SSOP | HLA-A, -B, -C and -DRB1 | 3,857 | RR | | 9 |
| Ann Woolfrey (2011) | BBMT | USA | 1999-2006 | PCR-SBT, PCR-SSOP | HLA-A, -B, -C and -DRB1 | 1,933 | RR | | 7 |
| Meerim (2011) | KJH | Asia | 2003-2009 | PCR-SSP | HLA-A, -B, -C and -DRB1 | 142 | HR | | 4 |
| John Horan (2012) | Blood | Asia  America | 1995-2007 | PCR-SBT, PCR-SSOP | HLA-A, -B, -C and -DRB1 | 663 | RR and OR | | 7 |
| Tso-Fu Wang (2012) | BBMT | Taiwan | 2005-2010 | Not available | HLA-A, -B, -C and -DRB1 | 693 | HR | | 4 |
| Wael Saber (2012) | Blood | USA | 2002-2006 | Not available | HLA-A, -B, -C and -DRB1 | 1,599 | RR and HR | | 8 |
| Carolyn Katovich Hurley (2013) | Blood | USA | 1988-2009 | PCR-SSP, PCR-SSOP | HLA-A, -B, -C and -DRB1 | 2,687 | HR | | 8 |
| Wael Saber  (2013) | Blood | USA | 2002-2006 | Not available | HLA-A, -B, -C and -DRB1 | 525 | RR | | 8 |
| Yoshinobu Kanda (2013) | BJH | Japan | 1993-2009 | Not available | HLA-A, -B, -C and -DRB1 | 3,003 | HR | | 7 |
| Joseph Pidala (2014) | Blood | USA | 1999-2011 | PCR-SSP, PCR-SSOP | HLA-A, -B, -C and -DRB1 | 8,003 | RR | | 9 |
| Marcelo A.Fernandez-Viña (2014) | Blood | USA | 1988-2009 | PCR-SBT, PCR-SSOP | HLA-A, -B, -C and -DRB1 | 7,349 | HR | | 9 |
| Junya Kanda (2015) | BBMT | Japan | 2000-2011 | PCR-SBT, PCR-SSP, PCR-SSOP | HLA-A, -B, -C and -DRB1 | 3,756 | HR | | 8 |
| Michael R.Verneris (2015) | BBMT | USA | 1999-2011 |  | HLA-A, -B, -C and -DRB1 | 2,588 | RR | | 8 |
| Craig Kollman (2016) | Blood | USA | 1988-2011 | PCR-SBT, PCR-SSOP | HLA-A, -B, -C and -DRB1 | 11,039 | HR | | 9 |

PCR, polymerase chain reaction; PCR-SBT, PCR-sequence-based typing; PCR-SSP, PCR-sequence-specific priming; PCR-SSOP, PCR-sequence-specific oligonucleotide probing; RFLP, restriction fragment length polymorphism; RSCA, reference strand conformation analysis; HR, hazard ratio; RR, relative risk, OR, odds ratio; NOS, Newcastle-Ottawa Scale; y, year.

**Table S3:** **Pooled analysis of one allele mismatches at individual HLA loci for post-transplantation end points.**

|  | **1 allele mismatches** | | | | | | |
| --- | --- | --- | --- | --- | --- | --- | --- |
| **Outcome** | **HLA locus** | **HR (95% CI)** | **P value** | **I^2^** | **N0** | **N1** | **N2** |
| **aGVHD (II-IV)** |  |  |  |  |  |  |  |
|  | HLA-A | 0.99 (0.67-1.44) | 0.93 | NA | 1 | 54 | 1,279 |
|  | HLA-B | 0.99 (0.69-1.44) | 0.97 | NA | 1 | 56 | 1,279 |
|  | HLA-C | 1.18 (0.90-1.56) | 0.229 | 59.1% | 2 | 259 | 6,058 |
|  | HLA-DRB1 | 1.60 (1.06-1.80) | 0.030 | NA | 1 | 34 | 1,279 |
|  | HLA-DQB1 | NA | NA | NA | NA | NA | NA |
|  | HLA-DPB1 | 1.30 (1.03-1.64) | 0.025 | 50.0% | 3 | 4,204 | 1,065 |
| **aGVHD (III-IV)** |  |  |  |  |  |  |  |
|  | HLA-A | 1.58 (1.27-1.96) | <0.001 | 0.0% | 3 | 354 | 5,085 |
|  | HLA-B | 1.69 (1.31-2.17) | <0.001 | 0.0% | 3 | 186 | 5,085 |
|  | HLA-C | 1.32 (1.04-1.69) | 0.024 | 55.5% | 4 | 879 | 9,864 |
|  | HLA-DRB1 | 1.33 (1.04-1.71) | 0.025 | 14.3% | 3 | 433 | 5,085 |
|  | HLA-DQB1 | NA | NA | NA | NA | NA | NA |
|  | HLA-DPB1 | 1.22 (1.08-1.38) | 0.002 | 0.0% | 3 | 4,741 | 1,907 |
| **cGVHD** |  |  |  |  |  |  |  |
|  | HLA-A | 0.99 (0.68-1.45) | 0.970 | NA | 1 | 54 | 1,279 |
|  | HLA-B | 0.87 (0.56-1.36) | 0.550 | NA | 1 | 56 | 1,279 |
|  | HLA-C | 0.98 (0.82-1.18) | 0.860 | 0.0% | 2 | 259 | 6,058 |
|  | HLA-DRB1 | 1.11 (0.68-1.84) | 0.670 | NA | 1 | 34 | 1,279 |
|  | HLA-DQB1 | NA | NA | NA | NA | NA | NA |
|  | HLA-DPB1 | NA | NA | NA | NA | NA | NA |
| **Engraftment** |  |  |  |  |  |  |  |
|  | HLA-A | 1.01 (0.87-1.16) | 0.915 | 0.0% | 1 | 187 | 1,599 |
|  | HLA-B | 0.69 (0.51-0.95) | 0.022 | 0.0% | 1 | 31 | 1,966 |
|  | HLA-C | 0.94 (0.82-1.07) | 0.358 | 0.0% | 1 | 524 | 1,966 |
|  | HLA-DRB1 | 0.92 (0.81-1.05) | 0.199 | 0.0% | 1 | 295 | 1,966 |
|  | HLA-DQB1 | NA | NA | NA | NA | NA | NA |
|  | HLA-DPB1 | NA | NA | NA | NA | NA | NA |
| **Relapse** |  |  |  |  |  |  |  |
|  | HLA-A | 0.79 (0.59-1.07) | 0.128 | 0.0% | 2 | 238 | 3,209 |
|  | HLA-B | 1.12 (0.75-1.69) | 0.576 | 0.0% | 2 | 88 | 3,209 |
|  | HLA-C | 0.88 (0.70-1.10) | 0.259 | 33.7% | 4 | 809 | 8,188 |
|  | HLA-DRB1 | 1.13 (0.90-1.43) | 0.293 | 0.0% | 2 | 334 | 3,209 |
|  | HLA-DQB1 | NA | NA | NA | NA | NA | NA |
|  | HLA-DPB1 | 0.78 (0.71-0.86) | <0.001 | 0.0% | 4 | 5,712 | 2,711 |
| **TRM** |  |  |  |  |  |  |  |
|  | HLA-A | 1.51 (1.28-1.78) | <0.001 | 0.0% | 4 | 412 | 6,558 |
|  | HLA-B | 1.59 (1.31-1.92) | <0.001 | 0.0% | 4 | 263 | 6,558 |
|  | HLA-C | 1.24 (1.07-1.45) | 0.005 | 39.1% | 7 | 996 | 11,526 |
|  | HLA-DRB1 | 1.25 (1.03-1.52) | 0.022 | 0.0% | 4 | 484 | 6,558 |
|  | HLA-DQB1 | 1.03 (0.59-1.81) | 0.910 | NA | 1 | 54 | 1,509 |
|  | HLA-DPB1 | 1.04 (0.74-1.44) | 0.830 | 58.4% | 3 | 4,204 | 1,025 |
| **Mortality** |  |  |  |  |  |  |  |
|  | HLA-A | 1.32 (1.16-1.51) | <0.001 | 0.0% | 4 | 412 | 6,560 |
|  | HLA-B | 1.37 (1.17-1.61) | <0.001 | 0.0% | 4 | 263 | 6,560 |
|  | HLA-C | 1.16 (0.99-1.37) | 0.067 | 60.5% | 7 | 996 | 11,528 |
|  | HLA-DRB1 | 1.26 (1.04-1.52) | 0.019 | 50.1% | 4 | 484 | 6,560 |
|  | HLA-DQB1 | 0.76 (0.48-1.21) | 0.250 | NA | 1 | 54 | 1,511 |
|  | HLA-DPB1 | 1.00 (0.90-1.12) | 0.962 | 41.3% | 3 | 4,741 | 1,907 |
| **DFS** |  |  |  |  |  |  |  |
|  | HLA-A | 1.34 (1.12-1.59) | 0.001 | 0.0% | 3 | 225 | 4,594 |
|  | HLA-B | 1.25 (1.06-1.47) | 0.009 | 0.0% | 3 | 232 | 4,594 |
|  | HLA-C | 1.09 (0.96-1.23) | 0.196 | 0.0% | 4 | 419 | 9,373 |
|  | HLA-DRB1 | 1.31 (1.10-1.57) | 0.003 | 0.0% | 3 | 189 | 4,594 |
|  | HLA-DQB1 | 0.79 (0.52-1.20) | 0.260 | NA | 1 | 54 | 1,511 |
|  | HLA-DPB1 | NA | NA | NA | NA | NA | NA |

N0, number of studies; N1, number of patients with a specific HLA locus mismatches; N2, number of patients as corresponding controls; NA, not available.

**Table S4:** **Pooled analysis of 1 allele or 1 antigen mismatches at individual HLA Loci for post-transplantation end points.**

|  | **1 allele or 1 antigen mismatches** | | | | | | |
| --- | --- | --- | --- | --- | --- | --- | --- |
| **Outcome** | **HLA locus** | **HR (95% CI)** | **P value** | **I^2^** | **N0** | **N1** | **N2** |
| **aGVHD (II-IV)** |  |  |  |  |  |  |  |
|  | HLA-A | 1.33 (0.94-1.89) | 0.106 | 20.8% | 3 | 203 | 1,678 |
|  | HLA-B | 1.46 (0.74-2.88) | 0.269 | 68.1% | 2 | 120 | 1,619 |
|  | HLA-C | 1.43 (0.90-2.28) | 0.128 | 0.0% | 2 | 164 | 399 |
|  | HLA-DRB1 | NA | NA | NA | NA | NA | NA |
|  | HLA-DQB1 | 0.89 (0.37-2.14) | 0.790 | NA | 1 | 44 | 340 |
|  | HLA-DPB1 | NA | NA | NA | NA | NA | NA |
| **aGVHD (III-IV)** |  |  |  |  |  |  |  |
|  | HLA-A | 1.46 (1.04-2.06) | 0.030 | NA | 1 | 136 | 1,279 |
|  | HLA-B | 2.22 (1.51-3.25) | <0.001 | NA | 1 | 73 | 1,279 |
|  | HLA-C | NA | NA | NA | NA | NA | NA |
|  | HLA-DRB1 | NA | NA | NA | NA | NA | NA |
|  | HLA-DQB1 | NA | NA | NA | NA | NA | NA |
|  | HLA-DPB1 | NA | NA | NA | NA | NA | NA |
| **cGVHD** |  |  |  |  |  |  |  |
|  | HLA-A | 1.12 (0.87-1.43) | 0.380 | NA | 1 | 136 | 1,279 |
|  | HLA-B | 0.94 (0.64-1.36) | 0.730 | NA | 1 | 73 | 1,279 |
|  | HLA-C | NA | NA | NA | NA | NA | NA |
|  | HLA-DRB1 | NA | NA | NA | NA | NA | NA |
|  | HLA-DQB1 | NA | NA | NA | NA | NA | NA |
|  | HLA-DPB1 | NA | NA | NA | NA | NA | NA |
| **Engraftment** |  |  |  |  |  |  |  |
|  | HLA-A | NA | NA | NA | NA | NA | NA |
|  | HLA-B | NA | NA | NA | NA | NA | NA |
|  | HLA-C | NA | NA | NA | NA | NA | NA |
|  | HLA-DRB1 | NA | NA | NA | NA | NA | NA |
|  | HLA-DQB1 | NA | NA | NA | NA | NA | NA |
|  | HLA-DPB1 | NA | NA | NA | NA | NA | NA |
| **Relapse** |  |  |  |  |  |  |  |
|  | HLA-A | 0.95 (0.67-1.36) | 0.790 | NA | 1 | 136 | 1,243 |
|  | HLA-B | 0.89 (0.55-1.44) | 0.640 | NA | 1 | 73 | 1,243 |
|  | HLA-C | NA | NA | NA | NA | NA | NA |
|  | HLA-DRB1 | NA | NA | NA | NA | NA | NA |
|  | HLA-DQB1 | NA | NA | NA | NA | NA | NA |
|  | HLA-DPB1 | NA | NA | NA | NA | NA | NA |
| **TRM** |  |  |  |  |  |  |  |
|  | HLA-A | 1.45 (1.20-1.76) | <0.001 | 0.0% | 4 | 385 | 3,151 |
|  | HLA-B | 1.57 (1.25-1.97) | <0.001 | 0.0% | 3 | 226 | 3,092 |
|  | HLA-C | 1.32 (1.09-1.60) | 0.004 | 0.0% | 3 | 531 | 1,908 |
|  | HLA-DRB1 | NA | NA | NA | NA | NA | NA |
|  | HLA-DQB1 | 1.19 (0.85-1.67) | 0.309 | 0.0% | 2 | 139 | 1,849 |
|  | HLA-DPB1 | NA | NA | NA | NA | NA | NA |
| **Mortality** |  |  |  |  |  |  |  |
|  | HLA-A | 1.32 (1.22-1.43) | <0.001 | 0.0% | 7 | 1,135 | 8,148 |
|  | HLA-B | 1.32 (1.14-1.53) | 0.016 | 36.6% | 6 | 553 | 8,030 |
|  | HLA-C | 1.33 (1.12-1.58) | 0.001 | 72.7% | 5 | 1,363 | 5,065 |
|  | HLA-DRB1 | 1.14 (0.94-1.48) | 0.183 | 0.0% | 3 | 254 | 4,995 |
|  | HLA-DQB1 | 1.00 (0.85-1.18) | 1.000 | 25.2% | 4 | 417 | 5,006 |
|  | HLA-DPB1 | NA | NA | NA | NA | NA | NA |
| **DFS** |  |  |  |  |  |  |  |
|  | HLA-A | 1.17 (1.07-1.37) | 0.041 | 0.0% | 2 | 318 | 2,754 |
|  | HLA-B | 1.31 (1.08-1.59) | 0.005 | 0.0% | 2 | 179 | 2,754 |
|  | HLA-C | 1.22 (1.05-1.42) | 0.010 | 0.0% | 1 | 367 | 1,511 |
|  | HLA-DRB1 | NA | NA | NA | NA | NA | NA |
|  | HLA-DQB1 | 1.02 (0.62-1.69) | 0.925 | 66.7% | 1 | 95 | 1,511 |
|  | HLA-DPB1 | NA | NA | NA | NA | NA | NA |

N0, number of studies; N1, number of patients with a specific HLA locus mismatches; N2, number of patients as corresponding controls; NA, not available.

**Table S5: Pooled analysis of 1 or 2 allele mismatches at individual HLA loci for post-transplantation end points.**

|  | **1 or 2 allele mismatches** | | | | | | | |
| --- | --- | --- | --- | --- | --- | --- | --- | --- |
| **Outcome** | **HLA locus** | **HR (95% CI)** | **P value** | | **I^2^** | **N0** | **N1** | **N2** |
| **aGVHD (II-IV)** |  |  |  |  | |  |  |  |
|  | HLA-A | 1.19 (1.08-1.31) | <0.001 | 0.0% | | 2 | 1,156 | 8,532 |
|  | HLA-B | 1.34 (1.20-1.50) | <0.001 | 0.0% | | 2 | 568 | 9,120 |
|  | HLA-C | 1.27 (1.18-1.37) | <0.001 | 0.0% | | 2 | 2,867 | 6,821 |
|  | HLA-DRB1 | 1.22 (1.10-1.36) | <0.001 | 0.0% | | 2 | 2,376 | 7,312 |
|  | HLA-DQB1 | 1.08 (0.98-1.19) | 0.143 | 0.0% | | 2 | 2,616 | 7,072 |
|  | HLA-DPB1 | 1.42 (1.28-1.57) | <0.001 | 53.8% | | 6 | 14,019 | 4,957 |
| **aGVHD (III-IV)** |  |  |  |  | |  |  |  |
|  | HLA-A | 1.36 (1.22-1.52) | <0.001 | 0.0% | | 4 | 1,408 | 10,588 |
|  | HLA-B | 1.32 (1.16-1.50) | <0.001 | 0.0% | | 4 | 852 | 11,073 |
|  | HLA-C | 1.41 (1.09-1.83) | 0.010 | 79.2% | | 4 | 3,106 | 8,502 |
|  | HLA-DRB1 | 1.22 (1.07-1.38) | 0.002 | 0.0% | | 3 | 2,636 | 8,875 |
|  | HLA-DQB1 | 1.09 (0.94-1.26) | 0.250 | 0.0% | | 2 | 2,616 | 7,072 |
|  | HLA-DPB1 | 1.24 (1.15-1.35) | <0.001 | 0.0% | | 4 | 12,685 | 4,259 |
| **cGVHD** |  |  |  |  | |  |  |  |
|  | HLA-A | 1.19 (0.99-1.43) | 0.065 | 49.8% | | 3 | 1,099 | 8,876 |
|  | HLA-B | 1.08 (0.95-1.23) | 0.233 | 0.0% | | 3 | 726 | 9,259 |
|  | HLA-C | 1.22 (1.05-1.42) | 0.010 | 37.5% | | 3 | 2,429 | 7,097 |
|  | HLA-DRB1 | 1.04 (0.81-1.33) | 0.752 | 69.2% | | 3 | 2,208 | 7,933 |
|  | HLA-DQB1 | 1.16 (1.03-1.29) | 0.010 | 0.0% | | 2 | 2,169 | 6,149 |
|  | HLA-DPB1 | 1.02 (0.94-1.09) | 0.677 | 1.3% | | 3 | 6,615 | 3,030 |
| **Engraftment** |  |  |  |  | |  |  |  |
|  | HLA-A | 0.93 (0.87-0.99) | 0.028 | 0.0% | | 2 | 1,008 | 8,393 |
|  | HLA-B | 1.08 (0.66-1.77) | 0.762 | 50.0% | | 2 | 699 | 8,717 |
|  | HLA-C | 0.95 (0.90-1.00) | 0.054 | 0.0% | | 1 | 2,321 | 6,636 |
|  | HLA-DRB1 | 0.95 (0.88-1.02) | 0.147 | 0.0% | | 2 | 2,246 | 7,326 |
|  | HLA-DQB1 | 0.91 (0.85-0.98) | 0.014 | NA | | 1 | 2,166 | 5,583 |
|  | HLA-DPB1 | NA | NA | NA | | NA | NA | NA |
| **Relapse** |  |  |  |  | |  |  |  |
|  | HLA-A | 1.02 (0.80-1.31) | 0.869 | 52.8% | | 2 | 912 | 6,331 |
|  | HLA-B | 1.04 (0.80-1.35) | 0.788 | 41.9% | | 2 | 435 | 6,808 |
|  | HLA-C | 0.70 (0.62-0.79) | <0.001 | 0.0% | | 2 | 2,122 | 5,121 |
|  | HLA-DRB1 | 0.96 (0.80-1.14) | 0.623 | 0.0% | | 2 | 1,764 | 5,479 |
|  | HLA-DQB1 | 1.02 (0.86-1.21) | 0.806 | 0.0% | | 2 | 1,928 | 5,315 |
|  | HLA-DPB1 | 0.72 (0.66-0.79) | <0.001 | 9.1% | | 5 | 6,779 | 3,529 |
| **TRM** |  |  |  |  | |  |  |  |
|  | HLA-A | 1.22 (0.80-1.86) | 0.360 | NA | | 1 | 121 | 1,509 |
|  | HLA-B | 1.62 (1.26-2.08) | <0.001 | NA | | 1 | 237 | 1,509 |
|  | HLA-C | 1.55 (1.09-2.21) | 0.015 | NA | | 1 | 106 | 1,509 |
|  | HLA-DRB1 | 1.68 (1.17-2.40) | 0.005 | NA | | 1 | 108 | 1,509 |
|  | HLA-DQB1 | 1.27 (0.88-1.82) | 0.200 | NA | | 1 | 107 | 1,509 |
|  | HLA-DPB1 | 1.05 (0.80-1.36) | 0.739 | 53.3% | | 3 | 6,367 | 1,065 |
| **Mortality** |  |  |  |  | |  |  |  |
|  | HLA-A | 1.36 (1.25-1.48) | <0.001 | 21.5% | | 5 | 1,529 | 12,099 |
|  | HLA-B | 1.28 (1.15-1.42) | <0.001 | 28.5% | | 5 | 1,089 | 12,584 |
|  | HLA-C | 1.20 (1.12-1.27) | <0.001 | 0.0% | | 5 | 2,972 | 9,519 |
|  | HLA-DRB1 | 1.14 (0.98-1.32) | 0.084 | 62.3% | | 4 | 2,744 | 10,386 |
|  | HLA-DQB1 | 1.12 (1.02-1.22) | 0.017 | 2.8% | | 3 | 2,723 | 8,583 |
|  | HLA-DPB1 | 1.04 (0.97-1.12) | 0.283 | 31.0% | | 5 | 12,733 | 4,281 |
| **DFS** |  |  |  |  | |  |  |  |
|  | HLA-A | 1.22 (0.94-1.59) | 0.140 | NA | | 1 | 121 | 1,511 |
|  | HLA-B | 1.33 (1.04-1.69) | 0.024 | NA | | 1 | 237 | 1,511 |
|  | HLA-C | 1.20 (0.93-1.55) | 0.150 | NA | | 1 | 106 | 1,511 |
|  | HLA-DRB1 | 1.38 (1.07-1.77) | 0.014 | NA | | 1 | 108 | 1,511 |
|  | HLA-DQB1 | 1.06 (0.81-1.37) | 0.690 | NA | | 1 | 107 | 1,511 |
|  | HLA-DPB1 | NA | NA | NA | | NA | NA | NA |

N0, number of studies; N1, number of patients with a specific HLA locus mismatches; N2, number of patients as corresponding controls; NA, not available.


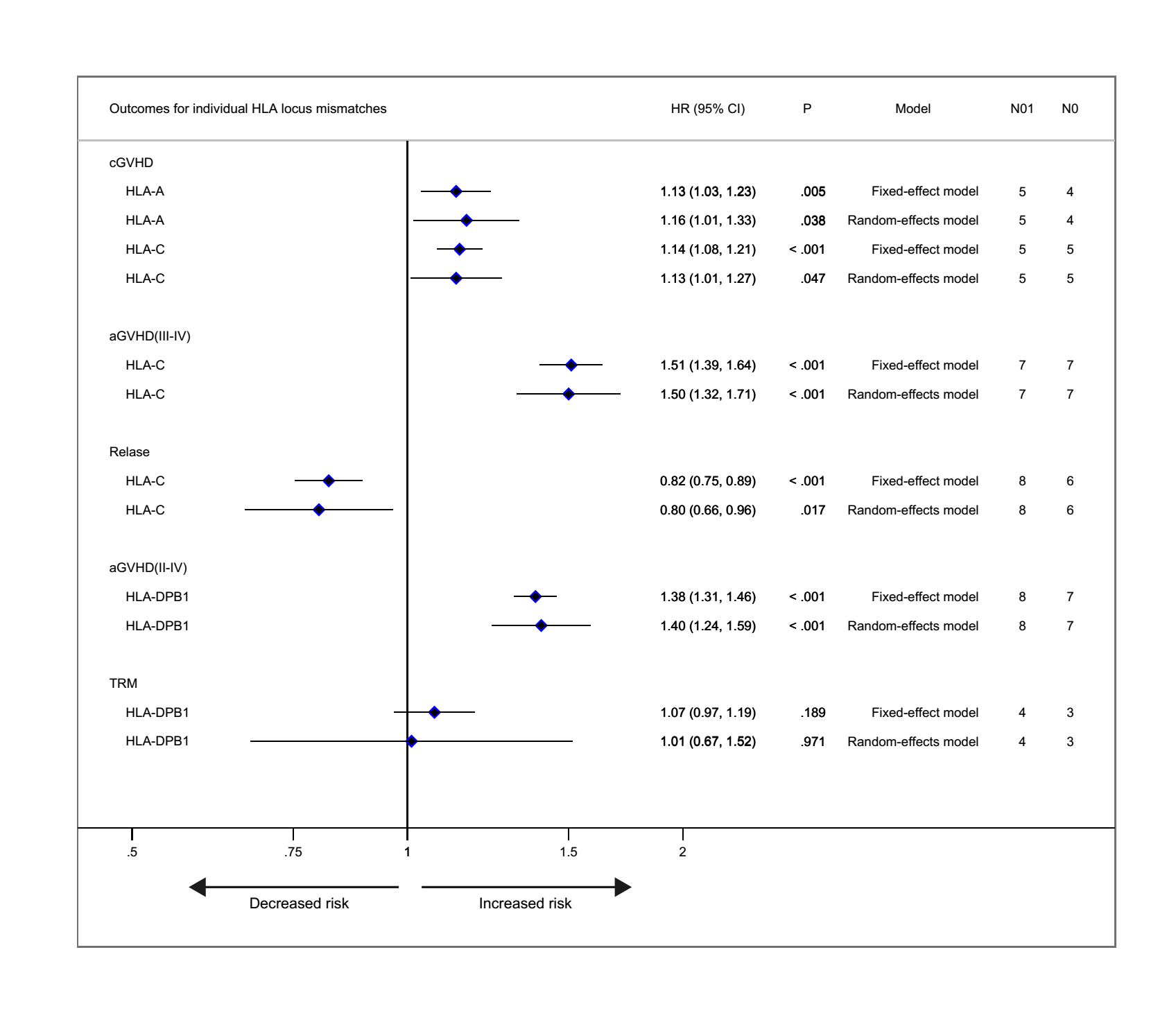


**Fig S1:** **Sensitivity analysis of pooled results with substantial heterogeneity for certain HLA locus mismatches.** Recalculation of pooled estimates using the trim and fill methods. N0, number of primary studies; N01, number of trim-fill studies
